# Performance Pressure as an Antecedent and Authentic Leadership as a Moderator of the Relationship Between Co-worker Undermining and Psychological Capital

**DOI:** 10.3389/fpsyg.2021.665362

**Published:** 2021-06-24

**Authors:** Eunmi Jang, Hyunkoo Kim

**Affiliations:** ^1^College of Business, Honam University, Gwangju, South Korea; ^2^Department of Business Administration, Soongsil University, Seoul, South Korea

**Keywords:** performance pressure, co-worker undermining, psychological capital, authentic leadership, conservation of resources theory

## Abstract

As a component of organizational aggression, co-worker undermining erodes the well-being of the victims and the sustainability of the organization. Drawing on conservation of resources theory, this study identified the negative impact of co-worker undermining on the victim’s psychological capital, and empirically examined the influence of performance pressure as an antecedent and of authentic leadership as a moderator to suggest approaches to minimize this negative impact. A total of 485 subordinate employees from 10 organizations in South Korea completed a questionnaire survey. To prevent common method bias, the survey was designed to recruit participants from multiple organizations and was conducted in two waves. First, the results revealed that performance pressure had a positive relationship with the perception of co-worker’s undermining. Second, this perception of co-worker undermining had a negative influence on the victim’s psychological capital. Third, authentic leadership had the moderating effect of decreasing the negative relationship between co-worker undermining and psychological capital. Furthermore, authentic leadership moderated the mediating relationship between the performance pressure and psychological capital through co-worker’s undermining. These findings suggest that the level of performance pressure should be managed in advance so as not to reach excessive levels and the psychological capital of victims should be preserved through authentic leadership to minimize the negative impact of co-worker undermining.

## Introduction

Social undermining has recently received attention as one form of organizational aggression that victimizes employees (Duffy et al., 2002). In particular, co-worker undermining may not only pose a psychological and physical threat to the victim (Aquino and Thau, 2009), but may also lead to serious conflicts within the organization, hindering its sustainability.

Many studies have been conducted to date on the negative consequences of co-worker undermining, but there is relatively little research on the antecedents of undermining. The role of leaders will likely be critical in minimizing the negative impact on victims in cases of co-worker undermining. However, few studies have analyzed what leadership styles can help alleviate the negative influence of co-worker undermining. Therefore, this study was conducted from the perspective of a sustainable workplace to provide data to facilitate pre-controlling and post-managing the negative effects of co-worker undermining.

There may be several predictors of victimization due to co-worker undermining, such as personality or other traits of the individual, but studies on organizational stressors are needed to elucidate the collective and implicit causes of co-worker undermining in the organization. In particular, performance pressure is a stressor caused by excessive task demands from the organization or supervisor, which leads employees to experience intense internal pressure (Mitchell et al., 2018) and strain (Lazarus and Folkman, 1984). Although performance pressure occurs frequently in most organizations, there has been little research on it, as opposed to other stressors. Moreover, no study has hitherto assessed whether employees who experience performance pressure may engage in negative interactions with their co-workers rather than with the organization or with supervisors. Therefore, this study empirically investigates whether performance pressure as an antecedent has significant influence on co-worker undermining.

Second, organizational aggression is known to cause negative emotions and attitudes in victims and degrade a victim’s well-being. It has been argued that this aggression damages the positive psychological resources of that employee (Martinko et al., 2013). To demonstrate this relationship, this study empirically examines the relationship between co-worker undermining and psychological capital (henceforth, “PsyCap”) of the victim. PsyCap refers to an individual’s positive psychological state, which acts as a personal resource that can predict the attitudes and behaviors expressed in stressful situations within an organization (Schaufeli and Bakker, 2004; Crawford et al., 2010; Christian et al., 2011). However, there are few studies on the relationship between negative organizational interactions and the victims’ PsyCap (Karatepe and Talebzadeh, 2016; Wu and Parker, 2016). Therefore, the current study focuses on co-worker undermining and empirically characterizes its relationship with victims’ PsyCap.

Third, this study draws on conservation of resources theory (Hobfoll, 1989; Halbesleben et al., 2014) to hypothesize that a victim’s lost PsyCap because of co-worker undermining could be regained by a positive leadership style. In particular, we focus on authentic leadership as one such positive leadership style (Luthans and Avolio, 2003; Avolio and Gardner, 2005; Walumbwa et al., 2007). Authentic leadership is a leadership style that can promote organizational performance and desirable organizational behaviors from employees based on authenticity. However, the effectiveness of this authentic leadership remains unclear, despite the positive reported aspects (Walumbwa et al., 2010, 2011). Therefore, additional research on specific factors of contextual difference is needed (Cooper et al., 2005; Yammarino et al., 2008) as are more empirical studies in diverse organizational context settings (Gardner et al., 2011; Petersen and Youssef-Morgan, 2018). Further, there exists an urgent need to study whether authentic leadership influences negative organizational situations.

Because the study assesses whether authentic leadership moderates the negative situation in which co-worker’s undermining lessens the PsyCap of the victim, its findings can contribute to more precisely establishing the role of authentic leadership in leadership theory.

In summary, this study investigates the influence of performance pressure as one of the antecedents of co-worker undermining. As a consequence of co-worker undermining, we examine the negative influence of undermining the PsyCap of the victim. In addition, we empirically investigate whether authentic leadership could decrease the negative impact of co-worker undermining on the PsyCap of the victim. We aim to elucidate implications that leaders could use to improve the sustainability of their organizations and employees by controlling the level of excessive performance demand and minimizing the negative impact of co-worker undermining through authentic leadership.

## Theory and Hypotheses Development

### Performance Pressure and Co-worker Undermining

Pressure is generally defined as any factor or combination of factors that increases the importance of performing well (Baumeister, 1984). Most organizations demand high performance from their employees (DeZoort et al., 2006), and such performance pressures put employees under stress to enhance their performance. In this way, employees potentially experience disadvantages if they fail to achieve the required performance level (Gutnick et al., 2012). Therefore, high performance pressure can lead employees to form negative emotions, attitudes, and behaviors, which degrade their well-being (Mitchell et al., 2018).

The stronger the performance pressure, the more employees need to justify their performance. According to social comparison theory, people continually evaluate their own traits or performance, but if they cannot find an objective basis for evaluation, they compare themselves to others around them (Festinger, 1954). Thus, employees under performance pressure try to evaluate their performance against their co-workers. When a performance discrepancy is recognized, namely that their performance is (or will be) worse than that of their co-workers, negative emotions, such as anxiety and envy are engendered. To relieve this stress, employees under performance pressure try to improve their performance. However, if they believe the scope for performance improvement is limited, they can be tempted to undermine the performance of their co-workers. In particular, forced distribution rating systems, currently in common use, tend to cause excessive internal competition and such performance pressure is more likely to trigger undermining of co-worker’s.

Therefore, when performance pressure is severe, there may be increased co-worker undermining behavior in real-world organizations, and under performance pressure, employees may also feel victimized through co-workers’ undermining. Accordingly, the following hypothesis was proposed:

***Hypothesis 1:*** Performance pressure is positively associated with co-worker undermining.

### Co-worker Undermining and PsyCap

Social undermining is one type of negative interaction that can occur within an organization (Aquino and Thau, 2009). It refers to behaviors intended to hinder a target person from creating and maintaining positive interpersonal relationships, achieving success at work, or maintaining a good reputation (Duffy et al., 2002). The two types of social undermining in business organizations are undermining by supervisors and by co-workers; however, the latter is more likely to occur due to performance pressure than the former, which requires additional performance.

By contrast, PsyCap is a positive psychological resource that consists of four sub dimensions: hope, resilience, self-efficacy, and optimism. This resource operates as an important source of internal motivation and is known to elicit desirable attitudes and behaviors among employees. Several studies have shown that PsyCap has a positive relationship with attitude to work (Avey et al., 2010) and job performance (Luthans et al., 2007; Peterson et al., 2012).

The perception of victimization due to the aggression of co-workers is likely to have a negative impact on the PsyCap of the victim. If the victim recognizes that co-worker’s undermining will diminish their chance of success in work and interpersonal relationships, the victim’s hope, resilience, self-efficacy, and optimism will decrease. This decline in PsyCap ultimately has a negative impact on the victim’s job performance and attitude. Accordingly, the following hypothesis was proposed:

***Hypothesis 2:*** Co-worker’s undermining is negatively associated with the subsequent PsyCap.

### Moderating Role of Authentic Leadership in the Relationship Between Performance Pressure and Co-worker Undermining

There are several studies on the relationship between leadership style and the perception of victimization by subordinates. The perception of victimization by subordinates tends to increase among leaders who are bureaucratic (Ashforth, 1997) or authoritarian (Coyne et al., 2003), those who do not share sufficient information with subordinates (Agervold and Mikkelsen, 2004), those who fail to resolve conflicts within their organizations (Hallberg and Strandmark, 2006), and laissez-faire leaders (Skogstad et al., 2007). Conversely, leaders with positive and open leadership styles may be able to decrease the perception of victimization following organizational aggression such as co-worker undermining.

Authentic leadership is a representative positive leadership style defined by four dimensions: self-awareness, internalized moral perspective, balanced processing of information, and relational transparency (Walumbwa et al., 2007). Authentic leaders make moral judgments based on their beliefs, regardless of social pressures (Taylor, 1992; Guignon, 2004), and reveal the relational characteristic of open communication with their subordinates (Walumbwa et al., 2007, 2011). Based on these characteristics, authentic leadership helps increase employees’ PsyCap (Avey et al., 2010).

The role of authentic leadership as a moderator of the negative relationship between co-worker undermining and the PsyCap of the victim can be explained by conservation of resources theory. The theory posits that individuals essentially pursue situations in which resources are sufficient and avoid situations in which resources can be lost (Hobfoll, 2001). As previously mentioned, the victim of co-worker undermining experiences severe stress. Cobb (1976) argued that the perception of social support by providing socio-emotional resources can be a moderating variable in such stressful situations. The behavior of the leader can be interpreted to correspond to that of the organization (Kang, 2019), leading employees to perceive that these direct supervisor actions are a form of social support in the organizational context (Eisenberger et al., 2002; Stinglhamber and Vandenberghe, 2003). Moreover, authentic leadership provides confidence in achieving the organization’s goal by the leader demonstrating their best ability, and encourages expectations and hopes for the future through transparent and fair communication (Bouckenooghe et al., 2014). Thus, the four positive dimensions of authentic leadership promote subordinates to form the positive PsyCap needed (Gardner et al., 2005).

Therefore, similar to social support, authentic leadership can act as a moderating variable that replenishes the PsyCap of victims lost owing to co-worker undermining. Although co-worker undermining leads to decreased PsyCap, the employees who perceive a high level of authentic leadership receive sufficient socio-emotional resources from the authentic leader such that the loss of PsyCap owing to co-worker undermining is relatively small. By contrast, employees who perceive a low level of authentic leadership will have an insufficient capability to cope with the stress caused by co-worker undermining. Therefore, authentic leadership will have the moderating effect of decreasing the strength of the negative relationship between co-worker undermining and PsyCap, thereby leading to the following hypothesis:

***Hypothesis 3:*** Authentic leadership moderates the relationship between co-worker undermining and the victim’s PsyCap, such that the association will be weaker when authentic leadership is high (versus low).

### Moderated Mediation Model Role of Authentic Leadership

With reference to the previously proposed hypothesis, this can be considered to be a moderated mediation model. Excessive performance pressure increases the perception of co-worker’s undermining and accordingly the victim’s PsyCap will be decreased. The perception of authentic leadership can moderate this indirect effect where performance pressure negatively affects PsyCap through co-worker’s undermining. In detail, the higher the level of their leader’s authentic leadership hat employees perceive, the more the negative effects of performance pressure on their PsyCap through co-worker’s undermining will be alleviated.

In other words, the indirect effect of performance pressure on PsyCap through co-worker’s undermining may vary depending on the perceived level of authentic leadership. Specifically, the perceived level of authentic leadership can moderate the influence of performance pressure on PsyCap which is mediated by co-worker’s undermining. Thus, the following hypothesis is established:

***Hypothesis 4:*** The perceived level of authentic leadership will moderate the mediating relationship between the performance pressure and PsyCap through co-worker’s undermining. This conditional indirect effect will be shown when the perceived level of authentic leadership is higher.

**FIGURE 1:**
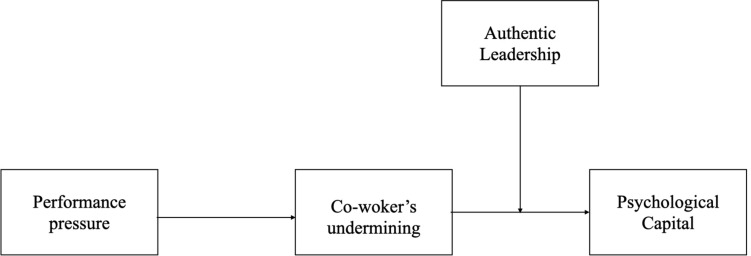
Theoretical model.

According to the above hypotheses, the research model was established, as shown in Figure 1.

## Materials and Methods

### Sample and Data Collection

To test our hypotheses, 485 employees who work at various companies in South Korea responded to an online survey. That is, to address potential sampling bias, data were collected using random sampling at two different time points. In this way, the limitations of cross-sectional research were addressed. Participants had the opportunity to complete the online survey during a 4-week period. Through this research design, we reduced concerns regarding common method bias (MacKenzie and Podsakoff, 2012). Specifically, 688 workers participated in our survey at the first time point and 490 employees at the second. Data from 485 responses were used in the final analysis. The characteristics of the sample are presented in Table 1.

**TABLE 1:** Descriptive characteristics of the sample.

Characteristic	Frequency	Percent
**Gender**		
* Male	247	50.9%
* Female	238	49.1%
**Age (years)**		
* 20–29	92	18.9%
* 30–39	215	44.3%
* 40–49	135	27.9%
* 50–59	43	8.9%
**Tenure (years)**		
* 1–4	249	51.4%
* 5–9	128	26.4%
* 10–14	66	13.5%
* over 15	42	8.7%
**Job level (rank)**		
* Assistant	254	52.3%
* Manager	109	22.5%
* Department Manager	54	20.6%
* Executive	22	4.5%

### Measures

The questionnaire used a five-point Likert scale, and the questionnaires originally constructed in English were translated into the Korean language (all questionnaire items used for the survey are provided in Appendix). We used a standard translation and back-translation procedure (Brislin, 1980) to ensure the reliability and validity of the research tool.

#### Performance Pressure

We measured employees’ perceptions of performance pressure using Mitchell et al.’s (2018) four-item scale. A sample item is “*The pressures for performance in my workplace are high*.” The resulting Cronbach’s α was 0.94.

#### Co-worker Undermining

We measured employees’ perceptions of co-worker’s undermining using Duffy et al.’s (2006) seven-item scale. A sample item is “*How often group members criticized them in front of other members/didn’t listen to them?*” The resulting Cronbach’s α was 0.97.

#### PsyCap

We measured employees’ perceptions of PsyCap using Luthans et al.’s (2007) 12-item scale. A sample item is “*I feel confident in representing my work area in meetings with management.*” The resulting Cronbach’s α was 0.92.

#### Authentic Leadership

We measured employees’ perceptions of authentic leadership using Walumbwa et al.’s (2007) ALQ 16-item scale. A sample item is “*My leader encourages everyone to speak their mind.*” The resulting Cronbach’s α was 0.96.

#### Control Variables

We included gender, age, education level of employees, status, and tenure as control variables because they may affect employee attitudes toward the organization (Tsui et al., 1992). Additionally, Woolley et al. (2011) found that gender could moderate the relationship between authentic leadership and positive organizational climate. The gender response option of “male” was coded as 0 and “female” as 1. Age, status, and tenure are likely to represent increased seniority over time, and knowledge or experience related to duties can affect members’ behavior when carrying out tasks (Wu and Parker, 2016). Age and tenure were measured in years. For status, the responses included “under assistant,” coded as 1; “under manager,” coded as 2; “under department manager,” coded as 3; and “over executive,” coded as 4. All control variables were collected at time point two.

### Statistical Analysis

All statistical analyses were performed using STATA 16.1. Before testing the hypotheses, we conducted a series of confirmatory factor analyses (CFAs) to examine the construct validities of the variables. To evaluate whether the model fit was acceptable, several goodness-of-fit indices were considered: comparative fit index (CFI), Tucker–Lewis index (TLI), root mean square error of approximation (RMSEA), and the standardized root mean square residual (SRMR). According to previous studies (Browne and Cudeck, 1992), to consider a model adequate, CFI and TLI should be greater than 0.90 and RMSEA below 0.06. Ordinary least-squares regression-based analysis was used to examine the direct and interaction effects. To examine the moderating effect, we mean centered the values of the independent variable and moderator and then created interaction terms using the centered variables. We also calculated the variance inflation factor (VIF) scores; the VIF scores of all variables were below 10 (Chatterjee et al., 2006).

## Results

### Descriptive Statistics

The means, standard deviations, and correlations of the variables are summarized in Table 2. There were significant correlations between performance pressure and each co-worker undermining and PsyCap. Co-worker undermining had a negative significant correlation with PsyCap but was not significantly correlated with authentic leadership. PsyCap was positively correlated with authentic leadership.

**TABLE 2:** Means, standard deviations, correlations, and reliabilities.

Variable	Mean	SD	1	2	3	4	5	6	7	8
(1) Gender	1.49	0.50	1							
(2) Age	37.46	8.37	−0.34**	1						
(3) Job level	2.64	1.46	−0.40**	0.66**	1					
(4) Tenure	2.73	1.17	−0.21**	0.45**	0.41**	1				
(5) PP	3.02	0.94	−0.09*	0.12**	0.21**	0.10*	(0.94)			
(6) Co-U	3.47	0.65	−0.14**	0.01	−0.01	−0.04	0.18**	(0.97)		
(7) PsyCap	1.77	0.94	−0.12*	0.32**	0.36**	0.19**	0.18**	−0.17**	(0.92)	
(8) AL	3.28	0.78	−0.01	0.08	0.10*	0.050	0.02	−0.06	0.43**	(0.96)

*N* = 485. *p < 0.05, **p < 0.01. () is a Cronbach’s alpha’s coefficient. PP = Performance Pressure, Co-U = Co-worker Undermining, AL = Authentic Leadership, PsyCap = Psychological Capital.

### Measurement Model

Table 3 presents the measurement model fit indices for the study variables. As previously mentioned, we conducted CFA using STATA 16.1 to examine the construct validities of the variables. As shown in Table 3, the fit indices supported that the hypothesized four-factor model of performance pressure, co-worker undermining, PsyCap, and authentic leadership (χ^2^ = 2091.73, *df* = 685; RMSEA = 0.06; CFI = 0.91, TLI = 0.90) yielded a better fit to the data than the three-, two-, and one-factor models. These CFA results confirm the distinctiveness of the four study variables for subsequent analyses.

**TABLE 3:** Chi-square difference tests and fit statistics for alternative measurement models.

Model	χ2	*df*	RMSEA	CFI	TLI	*Δdf*	Δχ2
4-Factor model ^*a*^	2091.73***	685	0.06	0.91	0.90	-	-
3-Factor model^b^	4418.22***	691	0.11	0.76	0.74	6	2325.49***
2-Factor model ^*c*^	6218.55***	701	0.13	0.68	0.66	10	1800.33***
1-Factor model ^*d*^	11194.04***	702	0.18	0.39	0.35	1	4975.49***

*N* = 485. ***p < 0.001. RMSEA = Root Mean Square Error of Approximation, CFI = Comparative Fit Index, TLI = Tucker-Lewis Index. PP = Performance Pressure Co-U = Co-worker Undermining, AL = Authentic Leadership, PsyCap = Psychological Capital.^*a*^4-Factor model = hypothesized model.^*b*^3-Factor model = AL and PsyCap merged.^*c*^2-Factor model = AL, PsyCap and PP merged.^*d*^1-Factor model = all variables merged.

### Hypothesis Testing

Hypothesis 1 posited that the perceptions of performance pressure would be positively associated with co-worker undermining. As shown in Model 2(Co-U) of Table 4, we found that the perceptions of performance pressure were significantly and positively related to co-worker undermining (*β* = 4.32, *p* < 0.001). Therefore, Hypothesis 1 was supported.

**TABLE 4:** Results of regressions testing Hypothesis 1 and Hypothesis 2.

Variable	Co-U		PsyCap	
	Model 1	Model 2	Model 1	Model 2
Gender	−3.60***	−3.62***	0.96	0.31
Age	0.37	0.54	2.63**	2.74**
Job level	–1.04	–1.77	4.47***	4.35***
Tenure	–1.37	–1.51	0.62	0.38
PP		4.32***		-
Co-U		-		−4.01***
*R*^2^	0.03	0.20	0.15	0.17
*ΔR^2^*	0.02	0.19	0.14	0.16
*F*	3.70**	6.80***	20.58***	20.19***

*n* = 485, **p < 001, ***p < 0.001. Entries are standardized regression coefficients. PP = Performance pressure, Co-U = Co-worker undermining, AL = Authentic leadership, PsyCap = Psychological capital.

Hypothesis 2 proposed that co-worker undermining would be negatively related to their PsyCap. As shown in Model 2 (PsyCap) of Table 4, we found that the perceptions of co-worker undermining were significantly and negatively related to their PsyCap (*β* = −4.01, *p* < 0.001). Therefore, Hypothesis 2 was also supported.

To test the moderating role of authentic leadership on the relationship between co-worker undermining and PsyCap (Hypothesis 3), we conducted hierarchical multiple regression analysis, as shown in Table 5. The interaction term (co-worker undermining × authentic leadership) was significant (*β* = 4.05, *p* < 0.001), as indicated in Table 5, Model 3. Accordingly, Hypothesis 3 was also supported.

**TABLE 5:** Results of hierarchical multiple regression testing Hypothesis 3.

Variables	PsyCap		
	Model 1	Model 2	Model 3
Gender	0.31	–0.02	0.25
Age	2.74**	2.73**	3.09**
Job level	4.35***	4.13***	4.25
Tenure	0.38	0.38	0.24
Co-U (A)	−4.01***	−3.82***	−4.82***
AL (B)		10.18***	1.39
A x B			4.05***
*R*^2^	0.17	0.32	0.34
*ΔR^2^*	0.17	0.31	0.33
*F*	20.19***	37.70***	35.70***

*n* = 485, **p < 001, ***p < 0.001. Entries are standardized regression coefficients. PP = Performance pressure, Co-U = Co-worker undermining, AL = Authentic leadership, PsyCap = Psychological capital.

**FIGURE 2:**
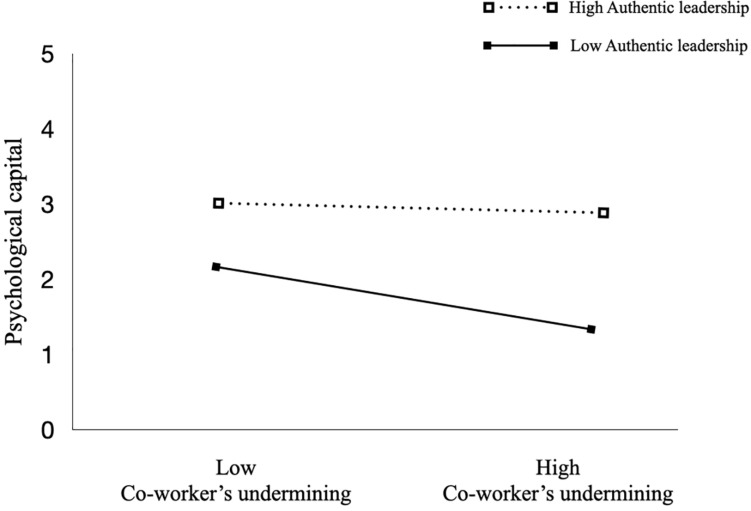
Moderating effect of authentic leadership.

This modulating effect of authentic leadership is shown in Figure 2, which illustrates that, although co-worker undermining and PsyCap are negatively related, employees who perceived greater authentic leadership tended to exhibit a smaller decrease in PsyCap than those who perceived lower levels of authentic leadership; that is, authentic leadership reduced the negative impact of co-worker undermining on PsyCap.

We also conducted a simple slopes test for the significant interaction. As predicted, the significance of the indices for high authentic leadership (*β* = −0.44, n.s) and low authentic leadership (*β* = −5.60, *p* < 0.001) supported Hypothesis 3.

Even though we identified the moderating effect by validation of the significance of the interaction terms by moderated multiple regression, we conducted additional testing utilizing the process macro model 1 suggested by Hayes (2017). We implemented 5,000 boot strapping sessions in addition, all variables were mean-centered.

The results of bootstrapping showed that the change in R^2^ according to the addition of interaction terms between co-worker’s undermining and authentic leadership was 0.01 (*p* < 0.01), which is statistically significant. The coefficient of the interactional terms was 0.11 (LLCI = 0.04, ULCI = 0.18), since zero is not included between LLCI and ULCI, the moderating effect of authentic leadership on the relationship between co-worker’s undermining and PsyCap was supported. Therefore, the significance of the moderating effect of authentic leadership has been re-verified.

To validate the moderating mediation model of Hypothesis 4, we utilized the conditional indirect effect analysis method suggested by Preacher et al. (2007) using model 14 of the process. And to verify the significance of each indirect effect depending on the perceived level of authentic leadership, 5,000 boot strapping sessions were performed. In addition, all variables were mean-centered for this analysis, and the results are shown as follows in Table 6.

**TABLE 6:** Conditional effect of Authentic leadership according to co-worker undermining and PsyCap.

Moderato r	Effect	Standard error	*p*-value	LLCI*	ULCI**
M-1SD (2.49)	−0.22	0.04	0.00	−0.31	–0.14
M (3.27)	−0.14	0.02	0.00	−0.19	–0.08
M+1SD (4.06)	−0.05	0.03	0.09	−0.12	0.01

*n* = 485, *LLCI = The lower limit in the 95% confidence section of the boot indirect effect; **ULCI = Upper limit within 95% confidence section of boot indirect effect.

Whether the moderated mediation effect is significant can be verified by testing the index of moderated mediation (Hayes, 2015). For moderated mediation effect verification, we performed a bootstrapping using mean ± standard deviation (M ± SD) to verify coefficient and statistical significance testing of indirect effects based on conditional values of authentic leadership. The moderated mediation effect of the average level of authentic leadership is −0.14, the effect of the group of the low level of authentic leadership perception (M-1 SD) is −0.22, and the effect of the group of high authentic leadership perception (M+1 SD) was −0.05. Therefore, as the level of authentic leadership perception increases, the effect of moderated mediation increases.

In particular, zero was not included between LLCI and ULCI in the groups with low authentic leadership perception (M-1 SD) and mean (M), but it was revealed that the effect was not significant in the groups with high authentic leadership perception (M+1 SD). Therefore, it is confirmed that authentic leadership perceived by employees moderates the mediation effect of the performance pressure on PsyCap through co-worker’s undermining and is regulated by the level of awareness of authentic leadership.

## Discussion

Co-worker undermining can damage sustainability of organizations and their employees. This study identified an antecedent and moderator that minimize the effect of organizational co-worker undermining. The results of this study can be summarized as follows. First, the study empirically demonstrated that excessive organizational performance pressure can lead to the negative action of undermining among employees.

Second, the perception of co-worker undermining reduced the PsyCap of the victimized employee. That is, the victim of co-worker undermining has less confidence in their ability, less hope and optimism that they can achieve the desired results, and less resilience to the stress experienced in the process of achieving the required performance level.

Third, if the employee perceives that the supervisor’s leadership is authentic, the loss of an employee’s PsyCap from co-worker undermining can be reduced. That is, authentic leadership is effective in the negative organizational context of co-worker undermining. To summarize, co-worker undermining has negative effects on a victim’s PsyCap; as such, excessive performance pressure should be controlled so that it does not cause co-worker undermining and leaders should exercise authentic leadership to minimize any negative influence of undermining.

The results of this study have the following theoretical and practical implications. First, this study focused on co-worker undermining as a form of organizational aggression and suggested two directions to reduce its negative impact. We considered performance pressure as an antecedent and empirically examined its relationship with co-worker undermining, which has not been studied to date. In particular, this study broadens the scope for further research by presenting both the organizational cause of intensive internal competition, namely performance pressures, and the psychological cause of victimization, namely stress from the possibility of failing to meet goals. These findings also have useful practical implications for organizations. Specifically, performance pressure may have the positive effect of improving short-term performance but a negative influence as well (Gardner, 2012), namely causing stress among employees, negative attitudes and behaviors of employees toward the organization or leader, as well as negative interactions among co-workers. Therefore, even if some degree of performance pressure is inevitable in organizations, it is necessary to ensure that it is not excessive.

Second, this study demonstrated empirically for the first time that co-worker undermining consumes the PsyCap of victims. This is consistent with Cassidy et al. (2014), who investigated the relationship between bullying, which is a similar form of organizational aggression, and PsyCap. This is further related to Duffy et al.’s (2006) argument that an individual who is socially undermined tends to perceive him- or herself as a victim of interpersonal injustice in the organization. By being undermined by co-workers within the same organization, the victim feels that they have experienced discrimination by the organization or supervisor (Tepper, 2000; Duffy et al., 2002), this stress eventually negatively affects their positive PsyCap. These findings show that organizations and leaders should maintain an equal level of exchange with all employees and try to resolve peer conflicts proactively to maintain a high level of positive PsyCap among employees.

Third, based on conservation of resources theory, we revealed that authentic leadership has a moderating effect that replenishes an employee’s PsyCap that was reduced by co-worker undermining. This is consistent with Cobb’s (1976) argument that the perception of social support can be the moderating variable in a stressful situation. Further, Salas Vallina et al. (2019) argued that leadership is a key contributor to individual ambidexterity, acting as the mechanism that balances the development of new knowledge and effective performance in clinical practice. Moreover, in situations in which the effectiveness of authentic leadership is questioned, its moderating effect, demonstrated in this study, will help to reinforce the theoretical basis of authentic leadership. Further, these results will also help rediscover the importance of the leaders’ roles in the context of negative interactions within the organization, especially the need for authentic leadership. In other words, if a leader honestly and authentically communicates with subordinates and shares detailed information fairly, an organizational climate of mutual cooperation will be created rather than competition or mutual antagonization. This climate will boost PsyCap, which drives future performance, even if there is undermining or conflict among co-workers. Therefore, in situations where negative interactions within the organization occur, leaders should be encouraged to exercise a higher level of authentic leadership.

In real world organizations, the most common pressure is a requirement for performance above a target level in a limited time period. Authentic leaders interact with their employees based on influence and encourage voluntary performative and desirable behaviors by role modeling. This process of role modeling takes considerable time and can conflict with the organization’s short-term performance pressures, resulting in a dilemma for authentic leaders. Therefore, leaders must manage a balance between the organization’s short-term performance needs and the development of employees, as argued by Salas Vallina et al. (2019).

### Limitations and Future Research

Despite the theoretical and practical implications, this study has some limitations. First, the data used in this study were all collected from the same respondents by self-report questionnaire, and there is concern about the common method bias. In order to prevent common method bias in research design, a longitudinal survey (2 times) was organized and conducted. The survey responses utilized in this study were collected from the same respondent twice with a time lag of 1 month. Nevertheless, we further conducted a single factor analysis suggested by Podsakoff et al. (2003) to verify whether Harman’s single factor test common method bias can be issued.

This test indicates that when all variables are inputted into the factor analysis at once, and the non-rotating factor analysis results are either aggregated as single factor or a single factor describes most of the covariances between the variables, then common method bias would occur. As results of the test, it is shown that a total of five factors were classified from factor analysis, and the single factor with the highest explanatory power is 26.14% of the total covariance. Therefore, common method bias can be considered not to be serious.

Second, our study only considered performance pressure. However, other antecedents may also cause co-worker undermining; these remain to be discovered and examined. Co-worker undermining is an organizational aggression that is often exposed to a superficial extent, but nevertheless affects the attitudes and behaviors of other employees negatively, while disrupting the cooperation within the organization and thereby hindering organization performance. To create sustainable organization performance, it would be desirable to identify the causes of negative behaviors and prepare solutions in advance, rather than reacting ex post facto. Therefore, similar studies need to be conducted on other challenging stressors, such as time pressure or role ambiguity, which can also appear while pursuing additional performance.

Third, effective leadership has a positive impact on the attitudes, behaviors, and performance of employees, and many studies have revealed that authentic leadership also has a positive relationship with work engagement (Walumbwa et al., 2010), OCB and job performance (Peterson et al., 2012). Although the current study investigated the effectiveness of authentic leadership as a moderator of the relationship between the perception of co-worker’s undermining and PsyCap, further studies are required because other moderators, such as negative social trends or proactive personality, could have different effects in diverse contexts. In particular, team-level study of authentic leadership and its influence is requested in the future, as it can give different implications from dyad-level research. Fourth, authentic leadership shares characteristics in common with other positive leadership styles, such as transformational or ethical leadership. In particular, these styles are exemplified by moral, ethical managers, and show characteristics of idealized influence in transformational leadership. However, despite these common characteristics, ethical and transformational leadership differ in that they exert not only indirect influence but also direct influence on their subordinates, while authentic leaders primarily exert indirect influence by role modeling. Therefore, it would be of interest for future studies to assess whether other positive leadership styles, such as transformational and ethical leadership, have the same moderating effect to alleviate the negative relationship between co-worker’s undermining and PsyCap.

Another limitation relates to the control variables in this study: only gender, age, status, and tenure were used as control variables. In future studies, educational background, employment type, job, and industry will need to be added as control variables to verify whether the analyzed relationships differ with respect to each control variable.

## Data Availability Statement

The original contributions presented in the study are included in the article/supplementary material, further inquiries can be directed to the corresponding author/s.

## Ethics Statement

Ethical review and approval was not required for the study on human participants in accordance with the local legislation and institutional requirements. The patients/participants provided their written informed consent to participate in this study.

## Author Contributions

EJ did the conceptualization, performed the methodology and software, validated and investigated the data, carried out the formal analysis, resources, and data curation, wrote the original draft, wrote, reviewed, and edited the manuscript, and visualized the data. HK supervised the data and carried out the project administration and funding acquisition. Both authors contributed to the article and approved the submitted version.

## Conflict of Interest

The authors declare that the research was conducted in the absence of any commercial or financial relationships that could be construed as a potential conflict of interest.

## Appendix

**Performance pressure (α = 0.94) Mitchell et al. (2018)**

(1) The pressures for performance in my workplace are high.

(2) I feel tremendous pressure to produce results.

(3) If I don’t produce at high levels, my job will be at risk.

(4) I would characterize my workplace as a results-driven environment.

**Co-worker undermining (α = 0.97) Duffy et al. (2006)**

(1) How often intentionally ignored them.

(2) How often gave them the silent treatment.

(3) How often went back on their word.

(4) How often look bad or slow you down.

(5) How often belittled them or their ideas.

(6) How often talked down to them.

(7) How often didn’t listen to them.

**Psychological capital (α = 0.92) Luthans et al. (2007)**

(1) I feel confident in representing my work area in meetings with management.

(2) I feel confident contributing to discussions about the organization’s strategy.

(3) I feel confident presenting information to a group of colleagues.

(4). If I should find myself in a jam at work, I could think of many ways to get out of it.

(5) Right now, I see myself as being pretty successful at work.

(6) I can think of many ways to reach my current work goals.

(7) At this time, I am meeting the work goals that I have set for myself.

(8) I can be “on my own,” so to speak, at work if I have to.

(9) I usually take stressful things at work in stride.

(10) I can get through difficult times at work because I’ve experienced difficulty before.

(11) I always look on the bright side of things regarding my job.

(12) I’m optimistic about what will happen to me in the future as it pertains to work.

**Authentic leadership (α = 0.96) Walumbwa et al. (2007)**

(1) My leader says exactly what he or she means.

(2) My leader admits mistakes when they are made.

(3) My leader encourages everyone to speak their mind.

(4) My leader tells you the hard truth.

(5) My leader displays emotions exactly in line with feelings.

(6)My leader demonstrates beliefs that are consistent with actions.

(7) My leader makes decisions based on his or her core values.

(8) My leader asks you to take positions that support your core values.

(9) My leader makes difficult decisions based on high standards of ethical conduct.

(10) My leader solicits views that challenge his or her deeply held positions.

(11) My leader analyzed relevant data before coming to a decision.

(12) My leader listens carefully to different points of view before coming to conclusions.

(13) My leader seeks feedback to improve interactions with others.

(14) My leader accurately describes how others view his or her capabilities.

(15) My leader knows when it is time to reevaluate his or her position on important issues.

(16) My leader shows he or she understands how specific actions impact others.

## References

[B1] Agervold M. Mikkelsen E. G. (2004). Relationships between bullying, psychosocial work environment and individual stress reactions. *Work Stress* 18 336–351. 10.1080/02678370412331319794

[B2] Aquino K. Thau S. (2009). Workplace victimization: aggression from the target’s perspective. *Annu. Rev. Psychol.* 60 717–741. 10.1146/annurev.psych.60.110707.163703 19035831

[B3] Ashforth B. E. (1997). Petty tyranny in organizations a preliminary examination of antecedents and consequences. *Can. J. Adm. Sci.* 14 126–140. 10.1111/j.1936-4490.1997.tb00124.x

[B4] Avey J. B. Luthans F. Smith R. M. Palmer N. F. (2010). Impact of positive psychological capital on employee well-being over time. *J. Occup. Health Psychol.* 15 17–28. 10.1037/a0016998 20063956

[B5] Avolio B. J. Gardner W. L. (2005). Authentic leadership development: getting to the root of positive forms of leadership. *Leadersh. Q.* 16 315–338. 10.1016/j.leaqua.2005.03.001

[B6] Baumeister R. F. (1984). Choking under pressure: self-consciousness and paradoxical effects of incentives on skillful performance. *J. Pers. Soc. Psychol.* 46 610–620. 10.1037/0022-3514.46.3.610 6707866

[B7] Bouckenooghe D. Zafar A. Raja U. (2014). How ethical leadership shapes employees’ job performance: the mediating roles of goal congruence and psychological capital. *J. Bus. Ethics* 129 251–264. 10.1007/s10551-014-2162-3

[B8] Brislin R. W. (1980). “Translation and content analysis of oral and written materials,” in *Methodology*, eds Triandis H. C. Berry J. W., (Boston, MA: Allyn and Bacon), 389–444.

[B9] Browne M. W. Cudeck R. (1992). Alternative ways of assessing model fit. *Soc. Methods Res.* 21 230–258. 10.1177/0049124192021002005

[B10] Cassidy T. McLaughlin M. McDowell E. (2014). Bullying and health at work: the mediating roles of psychological capital and social support. *Work Stress* 28 255–269. 10.1080/02678373.2014.927020

[B11] Chatterjee S. Hadi A. Price B. (2006). “Analysis of collinear data,” in *Regression Analysis by Example*, ed. Balding D. J. (Hoboken, NJ: Wiley), 143–174.

[B12] Christian M. S. Garza A. S. Slaughter J. E. (2011). Work engagement: a quantitative review and test of its relations with task and contextual performance. *Pers. Psychol.* 64 89–136. 10.1111/j.1744-6570.2010.01203.x

[B13] Cobb S. (1976). Social support as a moderator of life stress. *Psychosom. Med.* 38 300–314. 10.1097/00006842-197609000-00003 981490

[B14] Cooper C. D. Scandura T. A. Schriesheim C. A. (2005). Looking forward but learning from our past: potential challenges to developing authentic leadership theory and authentic leaders. *Leadersh. Q.* 16 475–493. 10.1016/j.leaqua.2005.03.008

[B15] Coyne K. S. Zhou Z. Thompson C. Versi E. (2003). the impact on health-related quality of life of stress, urge and mixed urinary incontinence. *BJU Int.* 92 731–735. 10.1046/j.1464-410X.2003.04463.x 14616456

[B16] Crawford E. R. Jeffery A. LePine Bruce Louis Rich (2010). linking job demands and resources to employee engagement and burnout: a theoretical extension and meta-analytic test. *J. Appl. Psychol.* 95 834–848. 10.1037/a0019364 20836586

[B17] DeZoort T. Harrison P. Taylor M. (2006). Accountability and auditors’ materiality judgments: the effects of differential pressure strength on conservatism, variability, and effort. *Account. Organ. Soc.* 31 373–390. 10.1016/j.aos.2005.09.001

[B18] Duffy M. K. Ganster D. C. Pagon M. (2002). Social undermining in the workplace. *Acad. Manage. J.* 45 331–351. 10.5465/3069350 3069350

[B19] Duffy M. K. Shaw J. D. Scott K. L. Tepper B. J. (2006). The moderating roles of self-esteem and neuroticism in the relationship between group and individual undermining behavior. *J. Appl. Psychol.* 91 1066–1077. 10.1037/0021-9010.91.5.1066 16953768

[B20] Eisenberger R. Stinglhamber F. Vandenberghe C. Sucharski I. L. Rhoades L. (2002). Perceived supervisor support: contributions to perceived organizational support and employee retention. *J. Appl. Psychol.* 87 565–573. 10.1037//0021-9010.87.3.565 12090614

[B21] Festinger L. (1954). A theory of social comparison processes. *Hum. Relat.* 7 117–140. 10.1177/001872675400700202

[B22] Gardner H. K. (2012). Performance pressure as a double-edged sword. *Adm. Sci. Q.* 57 1–46. 10.1177/0001839212446454

[B23] Gardner W. L. Avolio B. J. Luthans F. May D. R. Walumbwa F. (2005). Can you see the real me? A self-based model of authentic leader and follower development. *Leadersh. Q.* 16 343–372. 10.1016/j.leaqua.2005.03.003

[B24] Gardner W. L. Conliawe C. C. Davis K. M. Dickens M. P. (2011). Authentic leadership: a review of the literature and research agenda. *Leadersh. Q.* 22 1120–1145. 10.1016/j.leaqua.2011.09.007

[B25] Guignon C. B. (2004). *On Being Authentic.* New York, NY: Psychology Press.

[B26] Gutnick D. Walter F. Bernard A. N. Carsten K. W. Dreu D. (2012). Creative performance under pressure. *Organ. Psychol. Rev.* 2 189–207. 10.1177/2041386612447626

[B27] Halbesleben J. R. B. Neveu J. P. Underdahl S. C. P. Westman M. (2014). Getting to the “Cor”: understanding the role of resources in conservation of resources theory. *J. Manag.* 40 1334–1364. 10.1177/0149206314527130

[B28] Hallberg L. R. Strandmark K. S. (2006). Health consequences of workplace bullying: experiences from the perspective of employees in the public service sector. *Int. J. Qual. Stud. Health Well Being* 1 109–119. 10.1080/17482620600555664

[B29] Hayes A. F. (2015). An index and test of linear moderated moderated mediation. *Multivariate Behav. Res.* 50 1–22. 10.1080/00273171.2014.962683 26609740

[B30] Hayes A. F. (2017). Partial, conditional, and moderated moderated mediation: quantification, inference, and interpretation. *Commun. Monogr.* 85 4–40. 10.1080/03637751.2017.1352100

[B31] Hobfoll S. E. (1989). Conservation of resources: a new attempt at conceptualizing stress. *Am. Psychol.* 44 513–524. 2648906 10.1037//0003-066x.44.3.513

[B32] Hobfoll S. E. (2001). The influence of culture, community, and the nested−self in the stress process: advancing conservation of resources theory. *Appl. Psychol.* 50 337–421. 10.1111/1464-0597.00062

[B33] Kang S. W. (2019). Sustainable influence of ethical leadership on work performance: empirical study of multinational enterprise in South Korea. *Sustainability* 11 3101–3118. 10.3390/su11113101

[B34] Karatepe O. M. Talebzadeh N. (2016). An empirical investigation of psychological capital among flight attendants. *J. Air Transp. Manag.* 55 193–202. 10.1016/j.jairtraman.2016.06.001

[B35] Lazarus R. S. Folkman S. (1984). *Stress, Coping and Appraisal.* New York, NY: Springer Press.

[B36] Luthans F. Avolio B. J. (2003). “Authentic leadership development,” in *The Positive Organizational Scholarship*, eds Dutton J. E. Glynn M. A. Spreitzer G. (San Francisco, CA: Berrett-Koehler Publishers), 241–258.

[B37] Luthans F. Avolio B. J. Avey J. B. Norman S. M. (2007). Positive psychological capital: measurement and relationship with performance and satisfaction. *Pers. Psychol.* 60 541–572. 10.1111/j.1744-6570.2007.00083.x

[B38] MacKenzie S. B. Podsakoff P. M. (2012). Common method bias in marketing: causes, mechanisms, and procedural remedies. *J. Retail.* 88 542–555. 10.1016/j.jretai.2012.08.001

[B39] Martinko M. J. Harvey P. Jeremy R. B. Jeremy M. (2013). A review of abusive supervision research. *J. Organ. Behav.* 34 120–137. 10.1002/job.1888

[B40] Mitchell M. S. Baer M. D. Ambrose M. L. Folger R. Palmer N. F. (2018). Cheating under Pressure: a self-protection model of workplace cheating behavior. *J. Appl. Psychol.* 103 54–73. 10.1037/apl0000254 28805425

[B41] Petersen K. Youssef-Morgan C. M. (2018). The “Left Side” of authentic leadership: contributions of climate and psychological capital. *Leadersh. Organ. Dev. J.* 39 436–452. 10.1108/lodj-06-2017-0171

[B42] Peterson S. J. Walumbwaa F. O. Avolio B. J. Hannahc S. T. (2012). The relationship between authentic leadership and follower job performance: the mediating role of follower positivity in extreme contexts. *Leadersh. Q.* 23 502–516. 10.1016/j.leaqua.2011.12.004

[B43] Podsakoff P. M. Lee J. Y. Mackenzie S. B. (2003). Common method biases in behavioral research: a critical review of the literature and recommended remedies. *J. Appl. Psychol.* 88 879–903. 14516251 10.1037/0021-9010.88.5.879

[B44] Preacher K. J. Rucker D. D. Hayes A. F. (2007). Addressing moderated mediation hypotheses: theory, methods, and prescriptions. *Multivariate Behav. Res.* 42 185–227. 10.1080/00273170701341316 26821081

[B45] Schaufeli W. B. Bakker A. B. (2004). Job demands, job resources, and their relationship with burnout and engagement: a multi-sample study. *J. Organ. Behav.* 25 293–315. 10.1002/job.248

[B46] Skogstad A. Einarsen S. Torsheim T. Aasland M. S. Hetland H. (2007). The destructiveness of laissez-faire leadership behavior. *J. Occup. Health Psychol.* 12 80–92. 10.1037/1076-8998.12.1.80 17257068

[B47] Stinglhamber F. Vandenberghe C. (2003). Organizations and supervisors as sources of support and targets of commitment: a longitudinal study. *J. Organ. Behav.* 24 251–270. 10.1002/job.192

[B48] Taylor C. (1992). *The Ethics of Authenticity.* Cambridge, MA: Harvard University Press.

[B49] Tepper B. J. (2000). Consequences of abusive supervision. *Acad. Manag. J.* 43 178–190. 10.5465/1556375

[B50] Tsui A. S. Egan T. D. O’Reilly C. A. III (1992). Being different: relational demography and organizational attachment. *Adm. Sci. Q.* 37 549–579. 10.2307/2393472

[B51] Vallina A. S. Moreno-Luzon M. D. Ferrer-Franco A. (2019). The individual side of ambidexterity: do inspirational leaders and organizational learning resolve the exploitation-exploration dilemma? *Employee Relat.* 41 592–613. 10.1108/ER-02-2018-0050

[B52] Walumbwa F. O. Avolio B. J. Gardner W. L. Wernsing T. S. Peterson S. J. (2007). Authentic leadership: development and validation of a theory-based measure†. *J. Manag.* 34 89–126. 10.1177/0149206307308913

[B53] Walumbwa F. O. Luthans F. Avey J. B. Oke A. (2011). Authentically leading groups: the mediating role of collective psychological capital and trust. *J. Organ. Behav.* 32 4–24. 10.1002/job.653

[B54] Walumbwa F. O. Wang P. Wang H. Schaubroeck J. Avolio B. J. (2010). Psychological processes linking authentic leadership to follower behaviors. *Leadersh. Q.* 21 901–914.

[B55] Woolley L. Caza A. Levy L. (2011). Authentic leadership and follower development: psychological capital, positive work climate, and gender. *J. Leadersh. Organ. Stud.* 18 438–448. 10.1177/1548051810382013

[B56] Wu C. H. Parker S. K. (2016). The role of leader support in facilitating proactive work behavior. *J. Manag.* 43 1025–1049. 10.1177/0149206314544745

[B57] Yammarino F. J. Dionne S. D. Schriesheim C. A. Dansereau F. (2008). Authentic leadership and positive organizational behavior: a meso, multi-level perspective. *Leadersh. Q.* 19 693–707. 10.1016/j.leaqua.2008.09.004

